# Ameliorative effects of desert truffle *Terfezia claveryi* extract on *Hysterothylacium thalassini*- induced oxidative damage in C57BL/6 mice

**DOI:** 10.3389/fcimb.2026.1774323

**Published:** 2026-03-04

**Authors:** Mashael Alotaibi, Saleh Al Quraishy, Nada Almohawis, Simeon Santourlidis, Esam Al-Shaebi, Rewaida Abdel-Gaber

**Affiliations:** 1 Department of Zoology, College of Science, King Saud University, Riyadh, Saudi Arabia; 2 Department of Chemistry, College of Science, King Saud University, Riyadh, Saudi Arabia; 3 Epigenetics Core Laboratory, Institute of Transplantation Diagnostics and Cell Therapeutics, Medical Faculty, Heinrich-Heine University Düsseldorf, Düsseldorf, Germany

**Keywords:** anisakid nematodes, iNOS expression, oxidative stress, splenic pathology, *Terfezia claveryi*, zoonotic infection

## Abstract

**Background:**

Anisakid nematodes, particularly the third-stage larvae (L3) of *Hysterothylacium thalassini*, pose a significant zoonotic risk associated with the consumption of fish. Ingestion of infected fish can trigger oxidative stress, inflammatory responses, and tissue damage in mammalian hosts. The growing incidence of anisakid infections necessitates the exploration of natural therapeutic agents with antioxidant and immunomodulatory properties. *Terfezia claveryi*, a desert truffle widely consumed in Saudi Arabia, is recognized for its rich phytochemical composition and potential bioactivity, making it a promising candidate for mitigating oxidative damage caused by parasites.

**Aim:**

This study aimed to evaluate the therapeutic efficacy and antioxidant potential of *Terfezia claveryi* extract (TCE) against oxidative stress and splenic pathology induced by *H. thalassini* L3 infection in experimental mice.

**Methods:**

A methanol–water extract of *T. claveryi* was prepared and analyzed for its phytochemical constituents using Fourier Transform Infrared (FT-IR) spectroscopy and colorimetric assays. Male C57BL/6 mice were divided into control and infected groups, with infection induced using fresh, thermally processed, or frozen L3 larvae. TCE was administered orally at a dose of 250 mg/kg. Antioxidant status in spleen tissues was evaluated through enzymatic assays for catalase (CAT), superoxide dismutase (SOD), glutathione peroxidase (GPx), and reduced glutathione (GSH), while oxidative stress markers—malondialdehyde (MDA), hydrogen peroxide (H_2_O_2_), and nitric oxide (NO)—were quantified in spleen samples. Histopathological examination of spleen tissues was performed, and inducible nitric oxide synthase (iNOS) expression was analyzed by qPCR and ELISA.

**Results:**

Phytochemical screening of TCE revealed high phenolic content (46.72 ± 2.43 µg/ml), alongside measurable levels of flavonoids and tannins, confirming its strong antioxidant capacity. Infection with *H. thalassini* larvae caused pronounced splenic degeneration, congestion, and capsule thinning, accompanied by decreased antioxidant enzyme activities and GSH levels, and elevated oxidative markers (MDA, H_2_O_2_, NO). TCE treatment significantly restored antioxidant enzyme activities, reduced oxidative stress biomarkers, and improved splenic histoarchitecture. Moreover, iNOS gene and protein expression were markedly downregulated following TCE administration, indicating anti-inflammatory modulation.

**Conclusion:**

*T. claveryi* extract exhibits potent antioxidant and immunomodulatory effects that mitigate oxidative damage and inflammatory stress induced by *H. thalassini* infection in mice. Although the extract does not target the parasite burden directly, its ability to restore oxidant–antioxidant balance and modulate host immune responses suggests a valuable supportive or adjunctive role in reducing infection-associated tissue injury and immunopathology. These findings indicate that desert truffle–derived bioactive compounds may serve as natural complementary agents to limit oxidative and inflammatory complications associated with anisakid infections and warrant further investigation in combination with antiparasitic therapies.

## Introduction

Fish are an excellent source of high-quality protein and provide numerous essential vitamins and minerals vital for a balanced diet. However, they are susceptible to a variety of parasitic infections, which can pose significant health risks to humans who consume contaminated fish (Dela Cruz et al., 2022). Among these parasites, anisakid nematodes are particularly widespread worldwide (Utami et al., 2022). These nematodes belong to the families Anisakidae and Raphidascarididae (Fagerholm, 1991), with several genera—such as *Anisakis*, *Pseudoterranova*, *Contracaecum*, and *Hysterothylacium*—contributing significantly to the parasitic burden in fish. The genus *Anisakis* has received considerable attention due to its prevalence and associated health risks, but other genera are also ecologically and clinically important. Notably, *Hysterothylacium* comprises approximately 101 described species documented as parasites of marine fish, highlighting the diversity and complexity of host–parasite interactions in aquatic ecosystems (Menshawy et al., 2024). Understanding these relationships is essential for safeguarding the health of fish consumers and mitigating the risks of parasitic infections.

Anisakid parasites have attracted considerable attention in fisheries and public health due to their zoonotic potential and increasing incidence worldwide, particularly in regions with high consumption of raw or undercooked seafood (Fujisawa et al., 2025). Anisakidosis is reported most frequently in Japan, Europe, and coastal regions of the Americas, but its global prevalence is rising in parallel with the growing popularity of dishes such as sushi, sashimi, and marinated fish (Nagasawa, 2005). Human infection occurs through accidental ingestion of third-stage larvae (L3) present in infected marine fish or cephalopods. Although anisakid nematodes are unable to complete their life cycle in humans, larval penetration of the gastrointestinal mucosa can induce a wide range of clinical manifestations, including acute abdominal pain, nausea, vomiting, and, in some cases, intestinal obstruction. In addition, anisakid antigens may elicit strong immune responses, leading to hypersensitivity reactions, urticaria, or severe allergic symptoms, which can persist even after larval death (Audicana, 2022). Management of severe anisakidosis remains challenging, as treatment options are largely limited to endoscopic removal or symptomatic management, and currently available anthelmintic drugs have not demonstrated consistent efficacy in humans (Chai et al., 2024). These limitations underscore the need for continued research into alternative or complementary therapeutic approaches aimed at mitigating infection-associated pathology and host tissue damage.

Beyond fish and their associated parasitic risks, edible fungi—particularly mushrooms—have long been valued for both culinary and medicinal purposes. Mushrooms are celebrated worldwide for their distinctive flavors and aromas (Elkhateeb et al., 2019) and have historically been used to treat infections and other ailments (Jeitler et al., 2020). Among the diverse varieties of edible fungi, truffles stand out as some of the oldest and most nutritionally rich food sources, often considered comparable to traditional protein staples such as meat and fish (Janakat et al., 2005). Within this group, the dark brown *Terfezia claveryi* (Terfeziaceae) is particularly noteworthy. It is abundant and highly esteemed in Saudi Arabia, where it is popular among locals and gourmet chefs alike (Veeraraghavan et al., 2021). *T. claveryi* is especially valued for its nutritional profile, being rich in phenolic compounds—secondary metabolites with well-recognized preventive and therapeutic potential (Khlaif et al., 2021). Additionally, extracts from various fungi, including truffles, have demonstrated antimicrobial activity, highlighting their dual significance in dietary and medicinal contexts (İşlek et al., 2021).

Building on the nutritional and medicinal significance of *T. claveryi*, the present study aims to evaluate the potential therapeutic effects of its extract (TCE) against oxidative stress induced by *Hysterothylacium thalassini* third-stage larvae (L3) in experimental mice. Specifically, the study investigates whether TCE can mitigate oxidative alterations in key tissues, modulate antioxidant enzyme activity, and preserve cellular integrity under parasitic infection. By elucidating these protective effects, this research seeks to provide insight into the potential use of TCE as a natural intervention for parasite-induced oxidative damage.

## Materials and methods

### Collection of *Terfezia claveryi*

The brown desert truffles (*Terfezia claveryi*, locally known as *kamma*) were collected during the spring season from the Al-Mashaliyah Reserve in Tulayhah, Saudi Arabia (27°41′78″ N, 43°17′36″ E). The collection site is characterized by sandy, semi-arid soil typical of desert ecosystems. The species was taxonomically authenticated, and a voucher specimen (No. KSU-24710) was deposited in the Herbarium of the Botany Department, King Saud University, Riyadh, Saudi Arabia.

### Preparation of *Terfezia claveryi* extracts

The fruiting bodies of *T. claveryi* were thoroughly washed with distilled water to remove adhering debris, cut into small pieces, and shade-dried at room temperature to preserve thermolabile constituents. The dried material was then finely ground using a Hummer grinder (model ED-CG1400) to obtain a uniform powder. Following the procedure described by Souna et al. (2023), approximately 100 g of the powdered sample was macerated in a methanol/water mixture (70:30, v/v) for 2 hr under continuous agitation at room temperature, then refrigerated for 48 hr to enhance extraction efficiency. The resulting mixture was filtered through Whatman filter paper, and the filtrate was concentrated under reduced pressure at 40 °C using a rotary evaporator (Buchi, Switzerland). The concentrated extract was subsequently freeze-dried using a lyophilizer to obtain a dry powder, which was stored at –20 °C until reconstitution in distilled water for further analyses.

### Fourier-transform infrared spectroscopy

A small amount of TCE was mixed with potassium bromide (KBr) at a ratio of 1:99 (w/w) and thoroughly ground to achieve a uniform and homogeneous mixture. The resulting blend was finely pulverized to obtain a consistent particle size distribution suitable for spectroscopic analysis. FT-IR spectra were recorded using a Thermo Scientific NICOLET 6700 optical spectrometer (Waltham, USA), following the procedure described by Negrea et al. (2015). The spectra were obtained in the wavenumber range of 4000–400 cm^−1^ at a controlled temperature of 25 °C, and the characteristic absorption peaks were analyzed to identify the functional groups present in the extract.

### Total phenolic content (TPC)

TPC of the extract was determined using a modified Folin–Ciocalteu method as described by Bakchiche et al. (2022). Briefly, 0.1 mL of the extract was mixed with 2 mL of 2% (w/v) sodium carbonate (Na_2_CO_3_) solution and allowed to stand for 5 min. Subsequently, 0.1 mL of Folin–Ciocalteu reagent was added, and the reaction mixture was incubated in the dark at room temperature for 30 min. Absorbance was measured at 725 nm using a spectrophotometer against a reagent blank. A calibration curve was prepared using gallic acid as a standard over a concentration range of 10–200 μg/mL, showing linearity within this range. The limit of quantification (LOQ) was calculated based on the calibration curve parameters and was 12 μg/mL. TPC was calculated from the linear regression equation and expressed as milligrams of gallic acid equivalents (GAE) per gram of extract.

### Total flavonoid content (TFC)

TFC was determined using the aluminum chloride colorimetric method described by Shraim et al. (2021), with slight modifications. Briefly, 250 μL of the extract was mixed with 75 μL of 15% (w/v) sodium nitrite (NaNO_2_) solution and 1 mL of distilled water. After standing for 6 min, 75 μL of 10% (w/v) aluminum chloride (AlCl_3_) solution was added, followed by incubation for 5 min. Subsequently, 1 mL of 4% (w/v) sodium hydroxide (NaOH) was introduced, and the final volume was adjusted to 2.5 mL with distilled water. After 15 min, the absorbance was measured at 510 nm against a reagent blank using a spectrophotometer. A calibration curve was constructed using quercetin as a standard over a concentration range of 10–100 μg/mL, with good linearity observed. The limit of quantification (LOQ) for the assay was 10 μg/mL, calculated from the standard calibration curve. TFC was expressed as milligrams of quercetin equivalents (QUE) per gram of extract.

### Total tannin content (TTC)

TTC was quantified using the Folin–Denis method described by Schanderi (1970), with slight modifications. A stock solution of the extract was prepared at a concentration of 1 mg/mL. To 0.1 mL of this solution, 1 mL of distilled water and 0.5 mL of Folin–Denis reagent were added. The reaction mixture was then alkalinized with 1 mL of 15% (w/v) sodium carbonate (Na_2_CO_3_) solution and incubated in the dark for 30 min at room temperature. Absorbance was measured at 700 nm using a spectrophotometer. Tannin concentration was determined from a standard calibration curve constructed with tannic acid (20–120 μg/mL). All reagents except the extract or standard were included in the blank, with 0.1 mL of water used in place of the sample. Results were expressed as milligrams of tannic acid equivalents (TAE) per gram of extract.

### Fish and parasite collection

The greater lizardfish *Saurida tumbil* (Family: Synodontidae) (n = 30) was obtained from local fish markets in Jeddah City (Red Sea, Saudi Arabia). This species is known to commonly harbor third-stage (L3) anisakid larvae, particularly within the visceral cavity and digestive tract. The specimens were transported to the Parasitology Laboratory for examination. Following dissection, the visceral cavity and digestive tract of each fish were inspected macroscopically for L3 larvae in accordance with Abdel-Gaber et al. (2024). Recovered larvae were thoroughly washed several times in phosphate-buffered saline (PBS) to remove debris and then divided into three groups: (i) fresh L3 larvae, maintained at room temperature (23 °C) for 60 min; (ii) thermally treated L3 larvae, exposed to a 100 °C water bath for 60 min; and (iii) frozen L3 larvae, stored at −20 °C for 24 hr. Crude larval extracts were prepared by homogenizing twenty portions (approximately 10 larvae per mouse) of each treatment group. The homogenization was performed using microtube pestles, followed by sonication on ice at 100 W for five cycles of 30 sec each using an ultrasonic homogenizer (Iglesias et al., 1996). The resulting homogenates were centrifuged at 3000 rpm for 15 min at 4 °C, and the supernatant was collected and stored for subsequent experimental use.

### Mice animals

Male C57BL/6 mice (n = 40) were used in this study. Animals were obtained from the animal facility of the College of Pharmacy, King Saud University. At the start of the study, mice were 9–12 weeks old, weighing 20–25 g. They were housed in polypropylene cages, five mice per cage, in a controlled environment maintained at 23 ± 2 °C with a 12-hour light/12-hour dark cycle, and allowed to acclimate for one week before experimentation. Standard laboratory chow and water were provided *ad libitum*. Mice were randomly allocated into eight experimental groups (n = 5 per group), as detailed in **Table 1**. The study was conducted over 14 days and included two oral larval inoculations along with repeated administrations of TCE. Experimental infection was induced via oral delivery of third-stage (L3) larvae following the protocol described in **Table 1**. After infection, *Terfezia claveryi* extract (TCE) was administered orally to the respective treatment groups according to the group assignments specified in **Table 1**.

**Table 1:** Experimental grouping and treatment schedule of mice.

Experimental mice groups		Experimental design				
		Day 0 (first larval dosage)	Period between 0–7 days	Day 7 (second larval dosage)	Period between 7–14 days	Day 14
Control		Oral inoculation with dist. H_2_O	Oral inoculation with dist. H_2_O	Oral inoculation with dist. H_2_O	Oral inoculation with dist. H_2_O	Euthanization of mice
TCE			TCE (250 mg/kg) was orally given daily for 7 consecutive days		TCE (250 mg/kg) was orally given daily for 7 consecutive days	
Infected	Fresh L_3_	Oral inoculation with fresh L_3_	Oral inoculation with dist. H_2_O	Oral inoculation with fresh L_3_	Oral inoculation with dist. H_2_O	
	Thermal L_3_	Oral inoculation with thermal L_3_		Oral inoculation with thermal L_3_		
	Frozen L_3_	Oral inoculation with frozen L_3_		Oral inoculation with frozen L_3_		
Infected-TCE	Fresh L_3_	Oral inoculation with fresh L_3_	After 60 min of infection, TCE (250 mg/kg) was orally given daily for 7 consecutive days	Oral inoculation with fresh L_3_	After 60 min of infection, TCE (250 mg/kg) was orally given daily for 7 consecutive days	
	Thermal L_3_	Oral inoculation with thermal L_3_		Oral inoculation with thermal L_3_		
	Frozen L_3_	Oral inoculation with frozen L_3_		Oral inoculation with frozen L_3_		

### Sample collection

On day 14, mice were humanely euthanized using carbon dioxide (CO_2_) inhalation followed by cervical dislocation to ensure death, after which dissections were performed. The spleens were carefully excised, sectioned into small pieces, and preserved according to downstream applications: (i) in neutral buffered formalin (NBF) for histopathological analysis; (ii) in microtubes for oxidative status assays and stored at −80 °C; and (iii) in RNAlater for gene and protein expression studies, also stored at −80 °C.

### Histopathological examination

Spleen tissue samples were fixed in 10% NBF for 24 hr. The fixed tissues were then dehydrated through a graded series of ethanol, cleared in xylene, and embedded in paraffin wax. Sections of 5 µm thickness were cut using a microtome and processed for hematoxylin and eosin (H&E) staining following standard protocols. Stained sections were examined and imaged using an Olympus BX61 microscope (Tokyo, Japan). Capsule thickness was measured in five randomly selected fields per spleen for each mouse to assess histopathological changes.

### Biochemical investigations

Spleen tissues were homogenized at 10% (w/v) in ice-cold 0.1 M phosphate buffer (pH 7.4). The homogenates were centrifuged at 3000 rpm for 15 min at 4 °C, and the resulting supernatants were collected for biochemical analysis of oxidant–antioxidant parameters (Ahmed et al., 2025). Oxidative stress and antioxidant markers were quantified using commercial assay kits (Bio-Diagnostic Co., Egypt) according to the manufacturer’s instructions. Hydrogen peroxide (H_2_O_2_) determination was performed based on the presence of peroxidase (HRP), H_2_O_2_ reacts with 3,5-dichloro-2-hydroxybenzenesulfonic (DHBS) acid and 4-aminophenazone (AAP) to form a chromophore, measured spectrophotometrically at 520 nm (Aebi, 1984). Catalase (CAT, Catalogue no. CA 25 17) activity was determined based on the rate of decomposition of hydrogen peroxide (H_2_O_2_), measured spectrophotometrically at 240 nm (Aebi, 1984). Superoxide dismutase (SOD, Catalogue no. SD 25 21) activity was assessed by its ability to inhibit the reduction of nitroblue tetrazolium by superoxide radicals generated in the reaction system (Nishikimi et al., 1972). Glutathione peroxidase (GPx, Catalogue no. GP 2524) activity was measured through the enzymatic reduction of hydrogen peroxide using reduced glutathione as a substrate, coupled to the oxidation of NADPH (Paglia and Valentine, 1967). Reduced glutathione (GSH, Catalogue no. GR 25 11) levels were quantified based on its reaction with 5,5′-dithiobis-(2-nitrobenzoic acid) (DTNB), forming a yellow chromogen measured at 412 nm (Ellman, 1959). Nitric oxide (NO, Catalogue no. NO 25 33) production was estimated indirectly by measuring nitrite concentrations using the Griess reaction, which produces a chromophoric azo compound detectable at 540 nm (Green et al., 1982). Lipid peroxidation was evaluated by determining malondialdehyde (MDA, Catalogue no. MD 25 29) levels via the thiobarbituric acid reactive substances (TBARS) assay, based on the formation of a pink MDA–TBA adduct measured at 532 nm (Ohkawa et al., 1979). Hydrogen peroxide (H_2_O_2_, Catalogue no. HP 25) levels were quantified using a colorimetric method based on its reaction with a chromogenic substrate in the presence of peroxidase (Aebi, 1984). Absorbance readings were obtained using a SpectraMax^®^ 190 microplate reader, with data acquisition and analysis performed via SoftMax^®^ Pro software version 6.3.1. Each parameter was assessed in five samples per spleen for each mouse.

### Gene expression

Total RNA was extracted from spleen tissues using TRIzol reagent (Invitrogen, USA) following the manufacturer’s instructions. The isolated RNA was treated with DNase I (Applied Biosystems, Darmstadt, Germany) for at least 1 hr to remove genomic DNA contamination and subsequently reverse-transcribed into cDNA using a commercial reverse transcription kit (Qiagen, Hilden, Germany). Quantitative real-time PCR (qRT-PCR) was performed on an ABI Prism 7500HT sequence detection system (Applied Biosystems, Darmstadt, Germany) using SYBR Green PCR master mix (Qiagen, Hilden, Germany). The expression of inducible nitric oxide synthase (iNOS) was quantified using primers (Forward: 5’-TTGGAGCGAGTTGTGGATTTG-3’ and Reverse: 5’-GTAGGTGAGGGCTTGGCTGA-3’) obtained from Qiagen (Hilden, Germany). The thermal cycling program consisted of an initial denaturation at 95 °C for 10 min, followed by 40 amplification cycles of denaturation at 95 °C for 15 s, annealing at 60 °C for 30 s, and extension at 72 °C for 30 s. A final melt curve analysis from 65–95 °C was included to verify amplification specificity. Amplification and data analysis were performed using Applied Biosystems software version 3.1 (StepOne™, USA). Relative gene expression levels were calculated using the comparative Ct (2−ΔΔCT) method (Livak and Schmittgen, 2001), with β-actin serving as the internal reference gene with its related primers (Forward: 5’-GCTACAGCTTCACCACCACA-3’ and Reverse: 5’-AAGGAAGGCTGGAAAAGAGC-3’).

### Sandwich enzyme-linked immunosorbent assay (ELISA)

A quantitative assessment of iNOS protein levels in spleen tissues was performed using a mouse-specific ELISA kit (Bio-Diagnostic Co., Egypt) according to the manufacturer’s instructions to ensure accuracy and reproducibility. The assay has a limit of quantification of 5 pg/mL and a standard curve range of 5–500 pg/mL. Intra- and inter-assay coefficients of variation (CVs) were <8% and <10%, respectively, and the antibodies used were highly specific for iNOS, with minimal cross-reactivity against other nitric oxide synthase isoforms such as eNOS and nNOS. Optical densities (OD) were measured using a Bio-Rad iMark Microplate Reader (version SW 1.04.02.E), as described by Abdel-Gaber et al. (2025). iNOS concentrations were calculated by comparison to the standard curve and expressed in pg/mL.

### Statistical analysis

Experimental data were expressed as the mean ± standard deviation (SD), as SD reflects the variability among individual animals within each experimental group rather than the precision of the mean estimate. Prior to inferential analysis, the normality of data distribution was evaluated for each group using the Shapiro–Wilk test, and homogeneity of variances was verified. As the data did not significantly deviate from a normal distribution (p > 0.05), parametric statistical analyses were considered appropriate. Differences among experimental groups were analyzed using one-way analysis of variance (ANOVA), as the study design focused on comparing treatment effects within specific infection conditions rather than assessing interaction effects between independent factors. Statistical analyses were performed using SPSS software, version 18 (SPSS Inc., Chicago, Illinois, USA). A p-value ≤ 0.05 was considered statistically significant for all comparisons.

## Results

**Figure 1:**
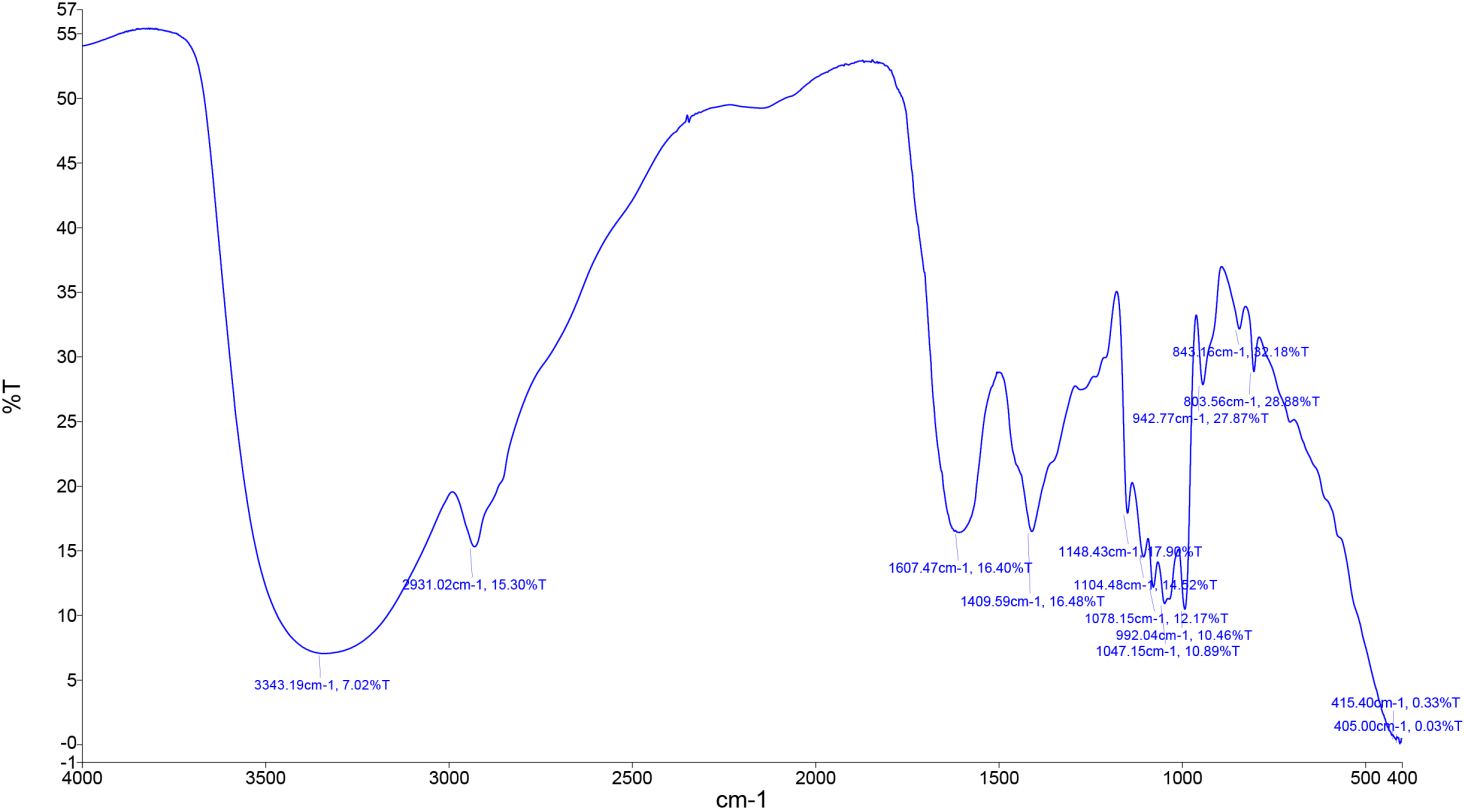
FT-IR of *Terfezia claveryi* extract (TCE) in an aqueous medium, showing the functional characteristic of the material.

The FT-IR spectrum revealed several characteristic absorption bands corresponding to functional groups and chemical classes (**Figure 1**; **Table 2**). A medium band at 3343 cm^−1^ indicated the presence of N–H stretching, characteristic of aliphatic primary amines. Peaks observed at 2931 cm^−1^ corresponded to C–H stretching vibrations of alkanes. A medium absorption at 1607 cm^−1^ was attributed to C=C stretching, suggesting the presence of conjugated alkenes. A strong band at 1409 cm^−1^ was assigned to S=O stretching, indicative of sulfate groups. Strong absorptions in the region between 1148 and 1078 cm^−1^ corresponded to C–O stretching in different functional groups, including tertiary alcohols (1148 cm^−1^), aliphatic ethers (1104 cm^−1^), and primary alcohols (1078 cm^−1^). A broad, strong band at 1047 cm^−1^ was characteristic of CO–O–CO stretching, consistent with anhydride functionalities. Additional strong signals at 992, 942, and 803 cm^−1^ were linked to C=C bending vibrations of alkenes. The spectrum also showed distinct features for halogenated compounds, including a strong C–Cl stretching band at 843 cm^−1^ and strong C–I stretching bands at 415 and 405 cm^−1^. Together, these data suggest the sample contains a mixture of functional groups, notably alkanes, alkenes, alcohols, ethers, sulfates, anhydrides, and halogenated compounds, with oxygenated functional groups (alcohols, ethers, anhydrides, and sulfates) being particularly well represented.

**Table 2:** FT-IR for *Terfezia claveryi* extract (TCE).

Absorption (cm^-1^)	Transmittance (%)	Appearance	Group	Compound class
3343.19	3.336026	medium	N-H stretching	aliphatic primary amine
2931.02	7.270826	medium	C-H stretching	alkane
1607.47	7.793566	medium	C=C stretching	conjugated alkene
1409.59	7.831583	strong	S=O stretching	sulfate
1148.43	8.506392	strong	C-O stretching	tertiary alcohol
1104.48	6.900157	strong	C-O stretching	aliphatic ether
1078.15	5.783396	strong	C-O stretching	primary alcohol
1047.15	5.175118	strong, broad	CO-O-CO stretching	anhydride
992.04	4.970774	strong	C=C bending	alkene
942.77	13.24431	strong	C=C bending	alkene
843.16	15.2925	strong	C-CI stretching	halo compound
803.56	13.72428	medium	C=C bending	alkene
415.40	0.156822	strong	C-I stretching	halo compound
405.00	0.014257	strong	C-I stretching	halo compound

Phytochemical profiling of the *Terfezia claveryi* extract (TCE) revealed three main classes of secondary metabolites—phenolics, flavonoids, and tannins. Among these, phenolic compounds, expressed as gallic acid equivalents, accounted for the largest share, reaching 46.72 ± 2.43 µg/ml. This high level of phenolics is especially notable because these compounds are well known for their potent antioxidant, anti-inflammatory, and antimicrobial properties, which could explain many of the biological activities linked to desert truffles. Flavonoids, measured as quercetin equivalents, were found at much lower concentrations (16.82 ± 0.96 µg/ml), suggesting that although they are present, they may have a more supporting role in the extract’s bioactivity. Tannins were also present at a significant level (24.20 ± 2.34 µg/ml), indicating they may contribute further to antioxidant effects and possibly anti-parasitic activity.

**Figure 2:**
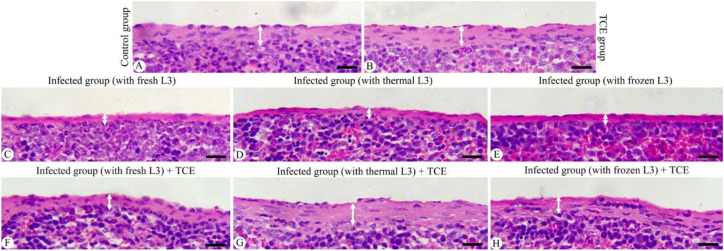
Histological alterations in the spleen capsule (white arrow) of mice infected with L_3_ larvae and treated with TCE. **(A)** Normal spleen capsule in an uninfected control (-TCE). **(B)** Normal spleen capsule in uninfected mice (+TCE). **(C)** Thinned capsule in spleen infected with fresh L_3_ (-TCE). **(D)** Thinned capsule in spleen infected with thermally treated L_3_ (-TCE). **(E)** Thinned capsule in spleen infected with frozen L_3_ (-TCE). **(F)** Moderately thickened capsule in spleen infected with fresh L_3_ and treated with TCE. **(G)** More pronounced capsule thickening in the spleen infected with thermally treated L_3_ and treated with TCE. **(H)** More pronounced capsule thickening in the spleen infected with frozen L_3_ and treated with TCE. Hematoxylin and eosin (H&E) staining, scale bar = 25 μm.

**Figure 3:**
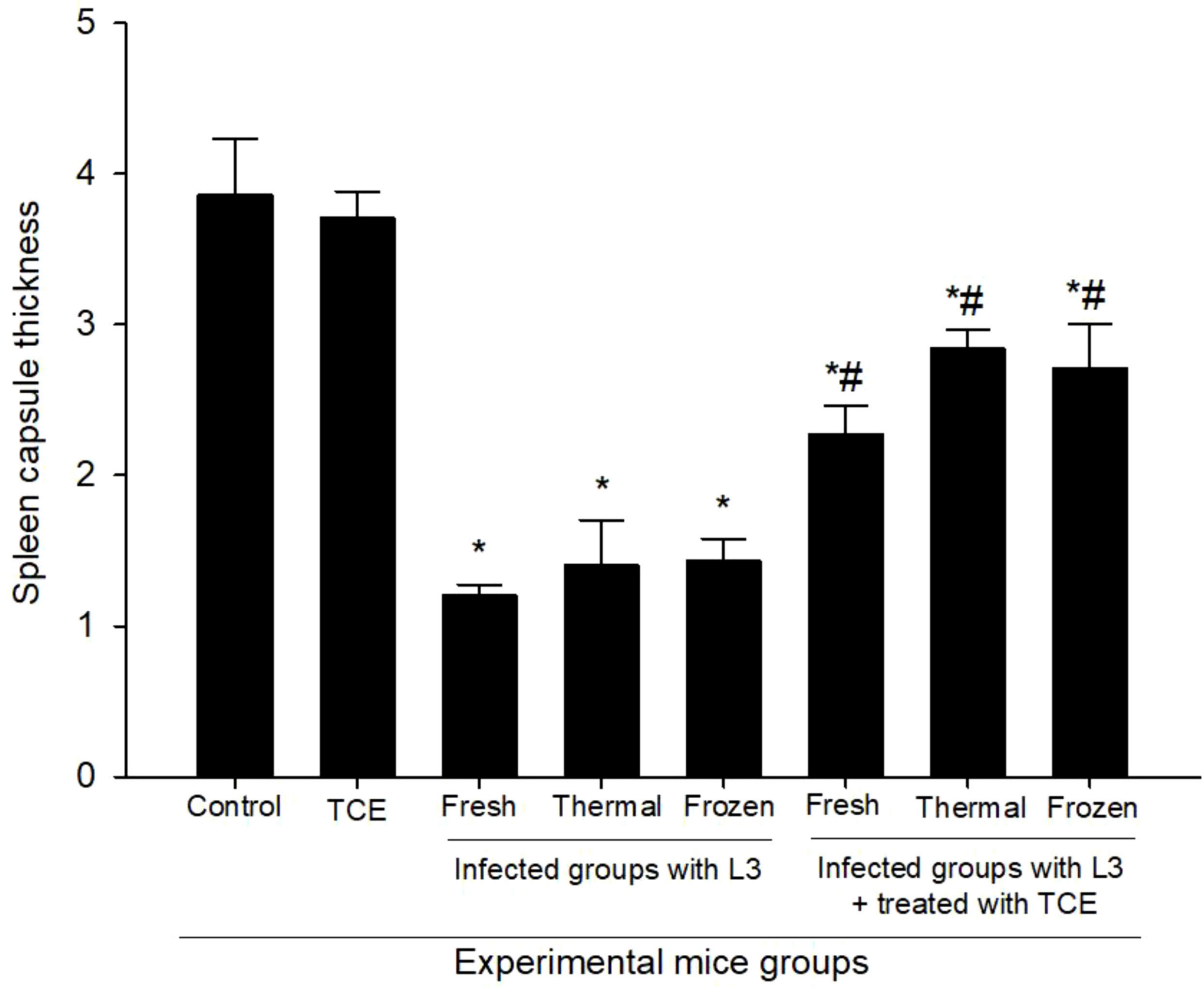
Changes in spleen capsule thickness in uninfected and infected groups with fresh, thermally, and frozen L_3_ larvae, as well as in infected groups administered TCE (250 mg/kg). * denotes a statistically significant difference relative to the control group; # denotes a statistically significant difference relative to the infected group with fresh L_3_ larvae.

The histological examination of the spleen revealed a marked reduction in tissue thickness following L_3_ infection compared to the normal control. In mice infected with fresh L_3_ larvae, the splenic thickness declined to 1.20 ± 0.07 μm, while infection with thermally processed L_3_ resulted in a slight increase but remained significantly reduced (1.40 ± 0.30 μm). Similarly, infection with frozen L_3_ yielded a thickness of 1.42 ± 0.15 μm, indicating that all infection types caused considerable splenic atrophy (**Figures 2A–E**). In contrast, administration of TCE produced a restorative and protective effect on splenic structure, significantly improving tissue thickness across all infected groups (**Figures 2F–H**). Specifically, in fresh L_3_-infected mice treated with TCE, splenic thickness rose to 2.27 ± 0.19 μm; in those infected with thermally processed L_3_, it further increased to 2.84 ± 0.13 μm; and in the group infected with frozen L_3_, TCE supplementation resulted in a thickening of splenic tissue, reaching 2.71 ± 0.30 μm. These results (**Figures 2**, **3**) clearly demonstrate both the degenerative impact of L_3_ infection on the spleen and the pronounced ameliorative effect of TCE treatment.

**Figure 4:**
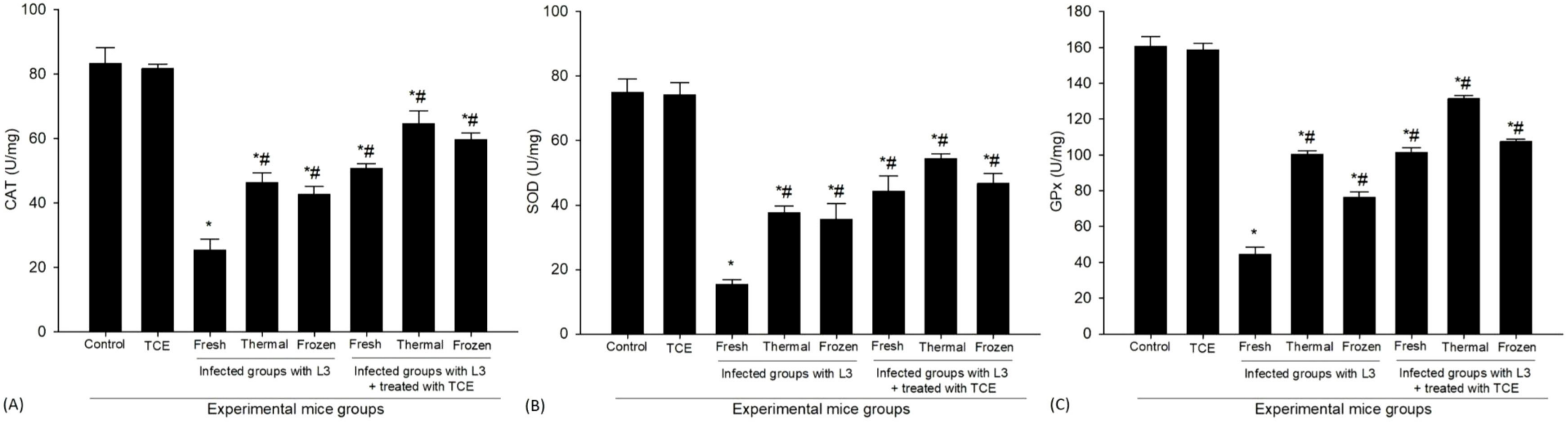
The levels of antioxidant enzymes of **(A)** Catalase (CAT), **(B)** Superoxide dismutase (SOD), and **(C)** Glutathione peroxidase (GPx) in the spleen of mice uninfected, infected with fresh, thermally, and frozen L_3_ larvae, as well as in infected groups administered TCE (250 mg/kg). * denotes a statistically significant difference relative to the control group; # denotes a statistically significant difference relative to the infected group with fresh L_3_ larvae.

Catalase (CAT) activity in the spleen tissue showed a pronounced suppression following L_3_ infection when compared to the normal control group. The control mice displayed a robust CAT activity of 83.33 ± 4.93 U/mg. However, infection markedly compromised this defense capacity. In mice exposed to fresh L_3_ larvae, CAT activity dropped drastically to 25.33 ± 3.51 U/mg, representing more than a 50% reduction relative to control values. Similarly, infection with thermally processed L_3_ produced a level of 46.33 ± 3.05 U/mg, while infection with frozen L_3_ resulted in 42.66 ± 2.51 U/mg, both significantly lower than control values. These reductions collectively highlight the detrimental effect of parasitic challenge on antioxidant enzyme functionality, suggesting that L_3_ infection disrupts the oxidative balance within splenic tissues. Conversely, supplementation with TCE produced a clear protective and restorative influence on CAT activity. Mice treated with TCE following infection with fresh L_3_ exhibited an improved CAT activity of 50.66 ± 1.52 U/mg, indicating partial recovery. The effect was even more pronounced in the thermally processed L_3_-infected group, where TCE treatment elevated CAT activity to 64.67 ± 4.04 U/mg. In the frozen L_3_-infected group, TCE also enhanced CAT levels to 59.67 ± 2.08 U/mg. These findings (**Figure 4A**) demonstrate that although infection strongly compromises antioxidant defenses, TCE supplementation significantly counteracts this effect by enhancing enzymatic activity, thereby contributing to the maintenance of redox homeostasis under parasitic stress.

The comprehensive analysis demonstrated a notable decrease in the levels of SOD in the groups of mice that were infected. Specifically, the concentration of SOD in these infected groups dropped significantly to an average of 15.33 ± 1.52 U/mg when the mice were exposed to fresh L_3_ larvae. Similarly, when exposed to thermally processed L_3_ larvae, the SOD concentration was measured at 37.67 ± 2.08 U/mg, while those infected with frozen L_3_ larvae exhibited a SOD level of 35.50 ± 4.95 U/mg. In contrast, the control group, which remained uninfected, maintained a baseline SOD level of 74.90 ± 4.19 U/gm. These findings strongly suggest that the infection significantly impairs the enzymatic activity of SOD. On a more positive note, treatment with TCE resulted in a significant recovery of SOD levels in the infected mice. Specifically, the SOD levels increased to 44.33 ± 4.73 U/mg in those infected with fresh L_3_ larvae. In cases involving thermally treated L_3_ larvae, the SOD levels have a further increase to 54.33 ± 1.52 U/mg, while the levels in the group infected with frozen L_3_ larvae reached 46.67 ± 3.21 U/mg. These results highlight the potential protective effects of TCE treatment against the decline in antioxidant defense mechanisms induced by the infection. This phenomenon is further illustrated in **Figure 4B**, which visually supports the hypothesis that TCE may enhance the antioxidant response in the face of infection.

The study revealed a significant reduction in the concentration of GPx in the infected mice group. The baseline GPx level in the control group was recorded at 160.66 ± 5.29 U/mg. However, in the infected mice, the GPx levels decreased to 44.33 ± 4.04 U/mg when subjected to fresh L_3_, to 100.33 ± 2.08 U/mg with thermally processed L_3_, and to 76.33 ± 3.05 U/mg in those infected with frozen L_3_. This marked decline indicates a significant compromise in the antioxidant defense mechanisms in these mice due to the infection. Subsequently, treatment interventions were administered, focusing on the impact of a dose of 250 mg/kg of TCE. Following this treatment, notable increases in GPx levels were recorded in the TCE-treated infected group. Specifically, the GPx concentrations rose to 101.33 ± 2.51 U/mg in mice infected with fresh L_3_, increased further to 131.33 ± 2.01 U/mg in those exposed to thermally treated L_3_, and reached 107.34 ± 1.52 U/mg in the mice infected with frozen L_3_. This data is represented in **Figure 4C**, illustrating the positive effects of treatment on the GPx concentrations.

**Figure 5:**
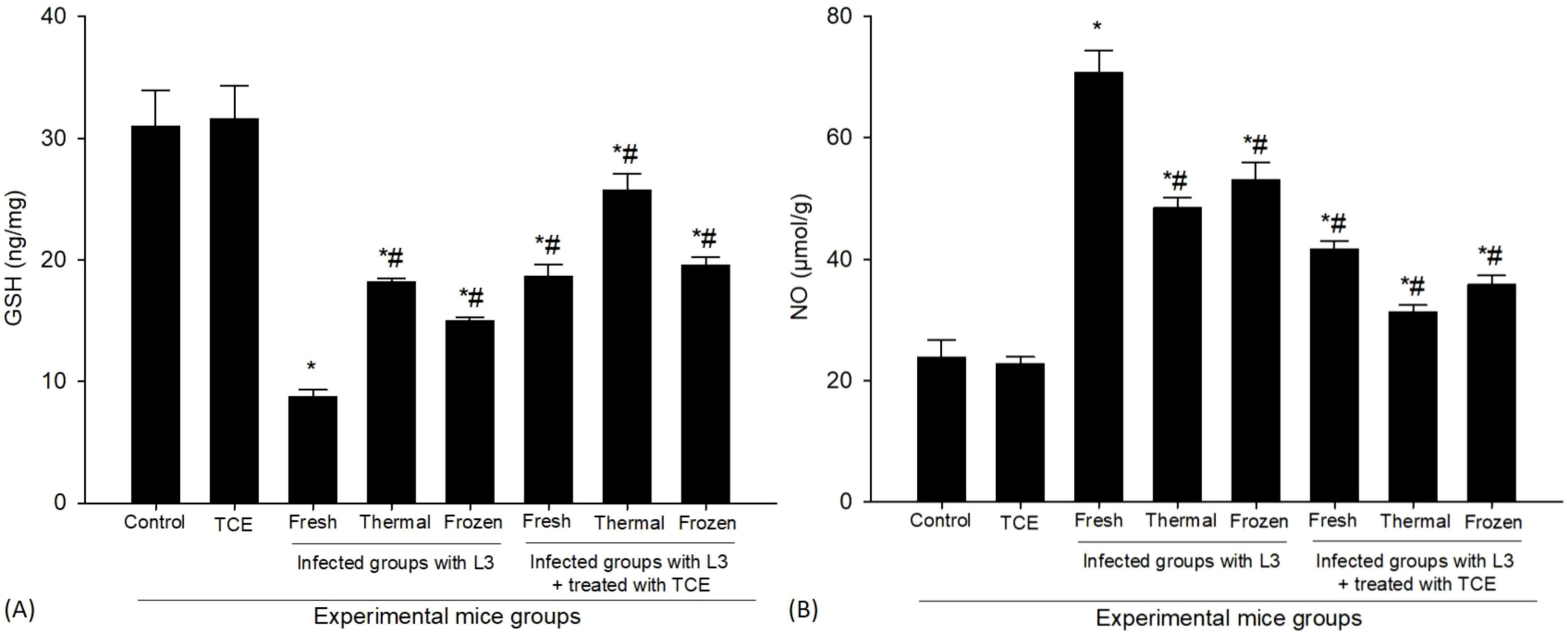
The levels of antioxidant markers of **(A)** Glutathione reduced (GSH) and **(B)** Nitric oxide (NO) in the spleen of mice uninfected, infected with fresh, thermally, and frozen L_3_ larvae, as well as in infected groups administered TCE (250 mg/kg). * denotes a statistically significant difference relative to the control group; # denotes a statistically significant difference relative to the infected group with fresh L_3_ larvae.

The concentration of GSH was markedly reduced in the group of mice that were infected. In the control group, the baseline GSH level was measured at 30.96 ± 2.95 ng/mg. However, upon exposure to fresh L_3_, this level decreased to 8.74 ± 0.55 ng/mg. Similarly, when the mice were subjected to thermally processed L_3_, GSH concentrations rose slightly but remained low at 18.16 ± 0.28 ng/mg. In cases where the mice were infected with frozen L_3_, GSH levels were also observed at 14.95 ± 0.29 ng/mg. In contrast, following treatment interventions, the levels of GSH in the mice that received a dosage of 250 mg/kg of TCE demonstrated a notable increase. Specifically, in the group treated with fresh L_3_ larvae, the GSH concentration increased to 18.60 ± 0.98 ng/mg, reflecting a partial recovery in antioxidant levels. When treated with thermally processed L_3_, the GSH level exhibited a significant increase to 25.70 ± 1.41 ng/mg. In the case of those infected with frozen L_3_, the GSH concentration was recorded at 19.55 ± 0.62 ng/mg. These findings suggest that the administration of TCE effectively upregulated the levels of GSH, thereby restoring a degree of antioxidant capacity in the mice that were infected. This recovery in GSH levels is represented in **Figure 5A**, reinforcing the therapeutic potential of TCE in combating the oxidative stress associated with parasitic infections.

The infection caused by L_3_ larvae led to a significant increase in NO levels in the spleen, showcasing some interesting variations based on the treatment of the larvae (**Figure 5B**). Specifically, when mice were exposed to fresh L_3_ larvae, the NO concentration surged to a remarkable 70.7 ± 3.76 µmol/g. This was contrasted by the response to thermally processed L_3_ larvae, which showed a slightly lower but still elevated NO level of 48.43 ± 1.69 µmol/g. Furthermore, those infected with frozen L_3_ larvae exhibited NO levels of 53.1 ± 2.81 µmol/g. These findings highlight that the state of the L_3_ larvae can influence the immune response, and the results indicate that the presence of L_3_ larvae significantly impacts NO production. In comparison, the control group, which did not experience any larval infection, showed a markedly lower NO level of 23.73 ± 2.96 µmol/g. Interestingly, when mice were treated with TCE, a notable decrease in NO levels was observed. Post-treatment results revealed that NO concentrations were reduced to 51.63 ± 1.30 µmol/g for those infected with fresh L_3_ larvae, illustrating a decrease from the pre-treatment levels. In the case of thermally processed L_3_, NO levels dropped significantly to 31.3 ± 1.12 µmol/g, and in those infected with frozen L_3_ larvae, NO was reduced to 35.73 ± 1.61 µmol/g. This reduction after TCE administration suggests that the treatment has the potential to counterbalance the elevated NO levels triggered by the infection. Such a decrease may help alleviate some oxidative stress and cellular damage associated with the presence of L_3_ larvae, indicating a therapeutic pathway for managing the oxidative stress resulting from this parasitic infection. This important observation is illustrated in **Figure 5B**.

**Figure 6:**
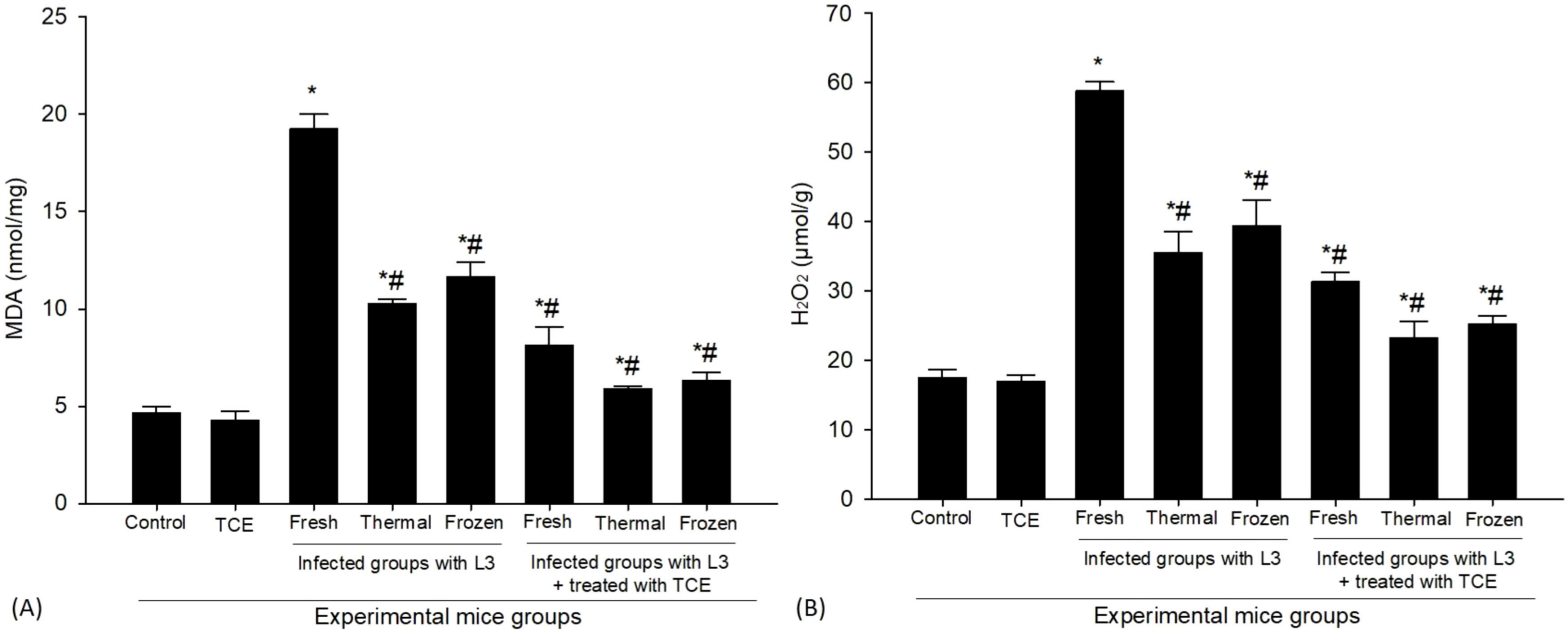
The levels of antioxidant markers of **(A)** Malonaldehyde (MDA) and **(B)** Hydrogen peroxide (H_2_O_2_) in the spleen of mice uninfected, infected with fresh, thermally, and frozen L_3_ larvae, as well as in infected groups administered TCE (250 mg/kg). * denotes a statistically significant difference relative to the control group; # denotes a statistically significant difference relative to the infected group with fresh L_3_ larvae.

The levels of MDA demonstrated a significant increase in the group of mice that were infected (**Figure 6A**). Specifically, the MDA concentrations were measured and revealed a significant rise from a baseline value of 4.64 ± 0.31 nmol/mg in the control group to considerably higher levels in the infected mice. When these infected mice were exposed to fresh L_3_ larvae, MDA levels surged to 19.23 ± 0.76 nmol/mg. Furthermore, the application of thermally processed L_3_ larvae resulted in MDA levels reaching 10.27 ± 0.20 nmol/mg, while infection with frozen L_3_ larvae led to MDA concentrations of 11.65 ± 0.74 nmol/mg. In contrast, treatment with TCE produced a significant reduction in MDA concentrations among the infected mice. Following the administration of TCE, MDA levels diminished notably to 8.14 ± 0.91 nmol/mg after exposure to fresh L_3_ larvae. Similarly, for those who received thermally processed L_3_, MDA concentrations were reduced to 5.88 ± 0.14 nmol/mg, and in the case of infections caused by frozen L_3_, MDA levels decreased to 6.30 ± 0.43 nmol/mg. These findings highlight the efficacy of TCE in alleviating oxidative stress, as evidenced by the marked decrease in MDA levels compared to the untreated infected group. This data is further illustrated in **Figure 6A**, which provides a visual representation of the significant impact of TCE treatment on oxidative stress levels in the context of infection.

In the context of our study, a clear distinction emerged when comparing the outcomes of the experimental group infected with L_3_ larvae against the control group. Notably, the presence of L_3_ larvae elicited substantial cellular damage, which was quantitatively assessed through the measurement of reactive oxygen species (ROS) levels, specifically H_2_O_2_. The results demonstrated a pronounced elevation in H_2_O_2_ concentrations within the spleen tissue of infected mice. For instance, spleens exposed to fresh L_3_ larvae recorded an H_2_O_2_ level of 58.5 ± 1.31 µmol/g. In contrast, those exposed to thermally processed L_3_ larvae showed slightly lower levels at 35.50 ± 3.04 µmol/g, while spleens infected with frozen L_3_ larvae exhibited intermediate H_2_O_2_ levels of 39.33 ± 3.78 µmol/g. These findings collectively underscore the oxidative stress inflicted on the spleen tissue as a result of the parasitic infection (**Figure 6B**). To explore potential therapeutic avenues, it was administered that treatment with TCE, an agent hypothesized to mitigate oxidative damage. The efficacy of TCE treatment was evident, as it significantly reduced the elevated levels of H_2_O_2_ associated with L_3_ infection. Post-treatment evaluations revealed that the H_2_O_2_ concentrations were reduced to 31.26 ± 1.41 µmol/g in the presence of fresh L_3_ larvae, to 23.23 ± 2.35 µmol/g for thermally processed L_3_, and to 25.16 ± 1.25 µmol/g for frozen L_3_ larval infections. This marked decrease reflects a successful intervention aimed at alleviating oxidative stress within the spleen tissue (**Figure 6B**). Such results not only highlight the detrimental oxidative damage prompted by parasitic infections but also emphasize the promising potential of TCE treatments in counteracting this oxidative stress and restoring cellular integrity.

**Figure 7:**
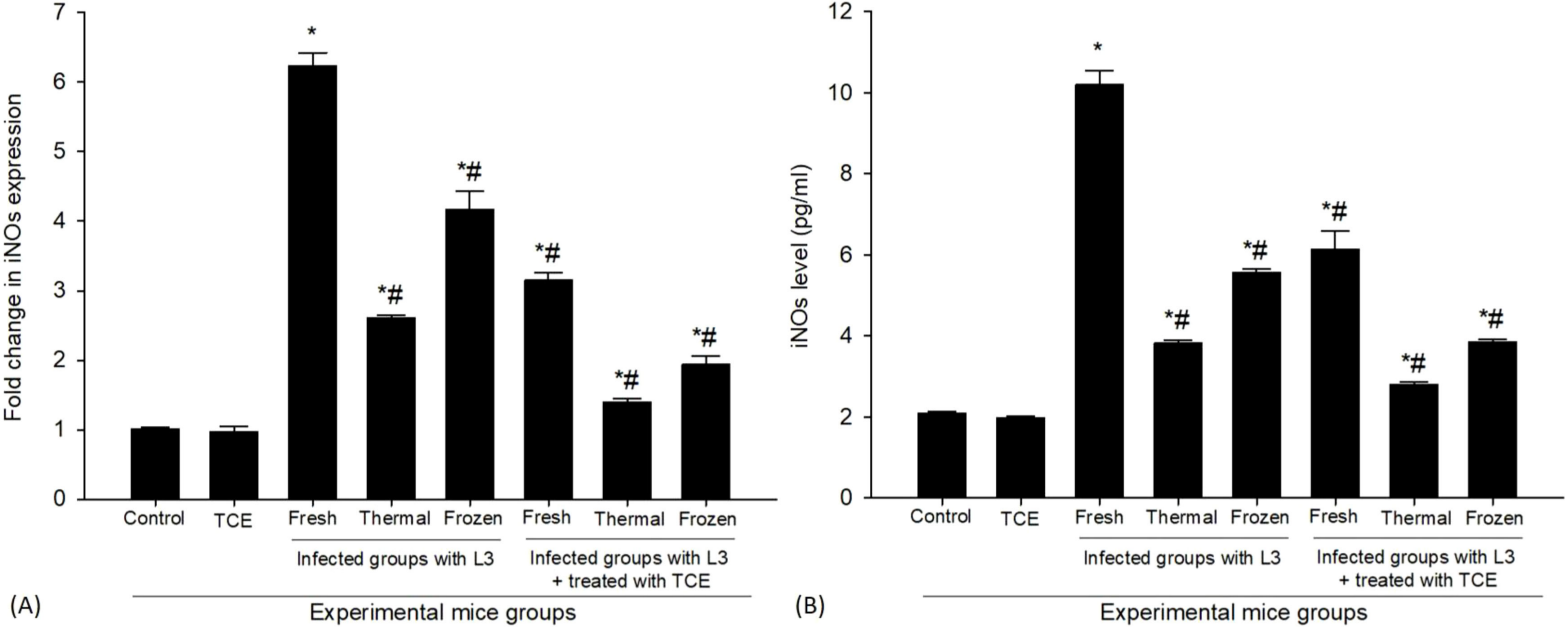
Effect of TCE on the level of iNOS along **(A)** mRNA expression and **(B)** protein expression in the spleen samples from the different experimental mouse groups. * denotes a statistically significant difference relative to the control group; # denotes a statistically significant difference relative to the infected group with fresh L_3_ larvae.

Following the infection with L_3_ larvae, we observed a significant increase in the mRNA expression levels of the iNOS gene (**Figure 7A**). Specifically, this expression level surged approximately 6.24-fold in mice exposed to fresh L_3_ larvae compared to the control group, which was standardized at a baseline expression level of 1.00-fold. In contrast, the mRNA levels increased by 2.60-fold when mice were exposed to thermally processed L_3_ larvae, and by 4.17-fold in those infected with frozen L_3_ larvae. These variations highlight the differing impacts of the larval conditions on the inflammatory response, as illustrated in **Figure 7A**. Following the administration of the treatment involving TCE, a marked decrease in mRNA expression of the iNOS gene was observed, indicating the treatment’s effectiveness in modulating the inflammatory response. Post-treatment measurements revealed that the iNOS gene expression levels dropped to approximately 3.15-fold for those exposed to fresh L_3_ larvae, 1.39-fold for those given thermally processed L_3_, and 1.93-fold for mice infected with frozen L_3_ larvae. This reduction in expression levels indicates that TCE plays a crucial role in mitigating the inflammatory processes initiated by the L_3_ larvae infection, thereby potentially alleviating the associated adverse effects (**Figure 7A**).

In our research, a comprehensive investigation was undertaken into the role of TCE within the context of L_3_-infection, placing emphasis on its impact on the production of iNOS. To accurately assess the levels of iNOS produced during the infection, ELISA methodologies were utilized, which are well-established techniques for quantifying specific proteins. Our findings, as depicted in **Figure 7B**, demonstrate that infection with the L_3_-parasite resulted in a substantial increase in iNOS levels. Specifically, measurements indicated that iNOS reached 10.19 ± 0.35 pg/ml when the parasites were in their fresh state, whereas levels were lower at 3.81 ± 0.08 pg/ml for thermally processed L_3_ and 5.55 ± 0.09 pg/ml for those infected with frozen L_3_, all in comparison to the control group, which exhibited normal, baseline levels of iNOS. This marked elevation in iNOS levels is a clear indicator of the immune response initiated by the presence of the L_3_-infection. Furthermore, treatment with TCE showcased a remarkable potential in reducing the elevated levels of iNOS triggered by the L_3_-infection. In the group treated with TCE, the iNOS levels showed a significant reduction to 6.12 ± 0.46 pg/ml in the presence of fresh L_3_, to about 2.80 ± 0.05 pg/ml when exposed to thermally processed L_3_, and to 3.84 ± 0.06 pg/ml for those infected with frozen L_3_. These values contrast with the elevated iNOS levels recorded in the infected group, as illustrated in **Figure 7B**. This observation suggests that TCE may not only influence the production of NO but also possesses the capability to modulate the immune response, potentially enabling a better management of the physiological effects resulting from L_3_-infection. The implications of these findings highlight the potential therapeutic benefits of TCE in enhancing immune function and controlling parasitic infections.

## Discussion

Parasitic infections generate substantial oxidative and inflammatory pressure within host tissues, resulting in alterations to cellular homeostasis and deterioration of organ architecture and performance (Reverte et al., 2022). Upon invasion, parasites commonly stimulate excessive production of reactive oxygen species (ROS) along with pro-inflammatory factors. This amplified response disrupts cellular stability, promotes oxidative damage to lipids, proteins, and DNA, and consequently impairs normal organ function (Pawłowska et al., 2023). Notably, the absence of significant differences among infections induced by fresh, thermally treated, and frozen larvae suggests that commonly used preventive processing methods may not completely mitigate parasite-associated biological effects, highlighting a potential One Health concern that warrants further investigation. In this context, TCE treatment was associated with improvements in oxidative stress–related parameters and inflammatory markers in the spleen of infected mice, suggesting a protective effect against parasite-induced oxidative imbalance. However, direct mechanisms such as ROS neutralization or membrane stabilization were not specifically assessed and would require further targeted experiments. It should be noted that all experiments were performed in male mice; therefore, potential sex-specific differences in immune and oxidative stress responses warrant further investigation.

FT-IR spectroscopic analysis of TCE confirmed the presence of multiple functional groups, including hydroxyl (–OH), ether (C–O–C), and sulfate (–SO_4_) moieties. These chemical signatures indicate the abundance of bioactive compounds such as phenolics, flavonoids, and tannins, which are well-documented for their antioxidant and immunomodulatory properties (Tel-Çayan et al., 2018). Phenolic and flavonoid molecules can donate hydrogen atoms and electrons to neutralize free radicals, chelate metal ions that catalyze ROS formation, and stabilize cellular membranes against peroxidative damage (Kruk et al., 2022). Tannins further contribute by modulating inflammatory signaling pathways, including NF-κB, thereby reducing cytokine overproduction (Karuppagounder et al., 2015). Phytochemical quantification revealed the predominance of phenolic compounds in TCE, corroborating previous reports on desert truffles where phenolic hydroxyl groups were identified as key contributors to ROS scavenging and redox homeostasis (Janakat and Nassar, 2010; Guetat et al., 2024).

Histological examination of splenic tissues from infected mice revealed pronounced structural alterations indicative of severe immune impairment. There was marked degeneration and disorganization of both the white pulp—responsible for lymphocyte proliferation and adaptive immune responses—and the red pulp, which is critical for blood filtration and erythrocyte turnover. Additionally, the splenic capsule appeared significantly thinner and less defined, reflecting tissue atrophy commonly associated with chronic parasitic infection and heightened oxidative stress (Abdel-Maksoud et al., 2017). These structural disruptions likely compromise the organ’s capacity to mount effective immunological responses, predisposing the host to further pathogen-induced damage (Liu et al., 2017). Remarkably, supplementation with TCE markedly restored splenic architecture, as evidenced by the re-establishment of well-organized white and red pulp regions and normalization of capsule thickness. This restorative effect suggests that TCE plays a crucial role in maintaining cellular integrity, promoting tissue regeneration, and preserving the functional microarchitecture of the spleen. Mechanistically, this protective action is likely mediated by the high content of phenolic and flavonoid compounds within TCE, which have been demonstrated to enhance the activities of endogenous antioxidant enzymes such as SOD, CAT, and GPx as reported by Kozarski et al. (2015). Concurrently, these bioactive compounds suppress lipid peroxidation and oxidative damage to membrane phospholipids, thereby stabilizing cellular and tissue structures under parasitic and oxidative stress conditions (Thiruvengadam et al., 2021; Muscolo et al., 2024). By restoring splenic structure and function, TCE supplementation may also improve systemic immune competence, as a structurally intact spleen is better able to coordinate lymphocyte activation, antibody production, and clearance of infected or damaged erythrocytes, consistent with Lewis et al. (2019). This suggests that the immunoprotective effects of TCE extend beyond localized tissue repair, potentially enhancing the host’s overall ability to resist parasitic infections and oxidative stress.

At the biochemical level, the infection-induced decline in critical antioxidant enzymes—including CAT, SOD, and GPx—alongside reduced levels of GSH, underscores the possible oxidative burden imposed on splenic tissues during *H. thalassini* infection. CAT is essential for decomposing H_2_O_2_ into water and oxygen (Kaushal et al., 2018), SOD catalyzes the dismutation of superoxide radicals into less reactive species (Wang et al., 2025), and GPx reduces lipid hydroperoxides and H_2_O_2_ (Ng et al., 2007), while GSH serves as a central intracellular antioxidant (Georgiou-Siafis and Tsiftsoglou, 2023), maintaining redox homeostasis. Pawłowska et al. (2023) reported that this enzymatic depletion reflects the overwhelming generation of ROS during parasitic infection, which surpasses the capacity of endogenous defense mechanisms, leading to oxidative damage to lipids, proteins, and nucleic acids, and ultimately compromising cellular and organ function. Treatment with TCE significantly restored the activities of these antioxidant enzymes and GSH levels, indicating a dual protective mechanism. First, the antioxidant constituents of TCE can directly scavenge free radicals, neutralizing ROS before they inflict cellular damage, as consistent with Sánchez (2017). Second, these bioactive compounds may modulate gene expression and signaling pathways that regulate the synthesis of antioxidant enzymes, thereby enhancing the endogenous defense system, consistent with Yu et al. (2024). The combined effect results in improved redox balance and mitigation of oxidative stress within splenic tissues. Comparable outcomes have been reported in studies employing plant extracts rich in phenolic compounds, which not only replenish antioxidant enzyme activities but also stabilize cellular membranes and reduce oxidative tissue injury (Shahat et al., 2014; Alqahtani et al., 2020; Villegas-Aguilar et al., 2024; Tüğen and Buruleanu, 2025). These findings collectively emphasize the role of TCE as a potent natural antioxidant capable of counteracting parasitic oxidative stress and preserving tissue integrity.

In parallel, the infection-induced elevation of MDA and H_2_O_2_ levels in splenic tissues of mice provides biochemical evidence of ongoing lipid peroxidation and oxidative membrane damage. MDA, a reactive aldehyde produced during the peroxidation of polyunsaturated fatty acids, serves as a widely recognized marker of cellular oxidative stress and reflects the extent of structural compromise to membrane lipids (Ayala et al., 2014). Similarly, elevated H_2_O_2_ levels indicate an accumulation of ROS capable of damaging proteins, nucleic acids, and mitochondrial membranes, thereby disrupting cellular homeostasis and impairing tissue function (Zorov et al., 2014). Notably, administration of TCE significantly reduced both MDA and H_2_O_2_ concentrations, demonstrating its potent ability to inhibit lipid peroxidation and reinforce antioxidant defenses. This protective effect is likely mediated by the rich phenolic and flavonoid content of TCE, which can directly scavenge ROS, donate hydrogen atoms to neutralize free radicals, and stabilize membrane structures by preventing oxidative chain reactions. These findings are consistent with previous studies that highlight the role of truffle-derived polyphenols in preserving membrane integrity and shielding cellular macromolecules from oxidative degradation, thereby contributing to overall cellular resilience and tissue homeostasis (Baldelli et al., 2025; Michalska et al., 2025; Yu et al., 2024).

The infection-induced upregulation of NO and iNOS represents a key component of the host’s innate immune response to parasitic invasion, functioning to limit pathogen proliferation and mediate signaling for immune cell recruitment (Tripathi et al., 2007). While basal NO production is protective, sustained or excessive NO generation during infection can be deleterious, leading to heightened oxidative stress, nitrosative damage, disruption of mitochondrial function, and impairment of cellular signaling pathways, all of which contribute to amplified tissue injury (Pacher et al., 2007; Meo et al., 2015; Iova et al., 2023). In the present study, treatment with TCE effectively downregulated both iNOS mRNA expression and protein levels in splenic tissues, suggesting an anti-inflammatory effect through modulation of NO synthesis. This regulatory action likely stems from the bioactive compounds in TCE—particularly phenolics, flavonoids, and truffle-derived polysaccharides—which are known to inhibit iNOS transcription and enzymatic activity by suppressing NF-κB activation, downregulating pro-inflammatory cytokine release, and interfering with other signaling pathways that amplify inflammation (Mamun et al., 2024). By controlling excessive NO production, TCE not only mitigates nitrosative and oxidative damage but also supports the restoration of tissue homeostasis, highlighting its potential as a natural immunomodulatory agent capable of attenuating parasite-induced inflammatory injury.

While this study provides compelling evidence for the antioxidant and immunomodulatory effects of TCE against *H. thalassini* infection, several limitations should be acknowledged. First, the investigation was confined to a murine model, which may not fully represent the complexity of host–parasite interactions in natural fish or human hosts. Second, the extract was evaluated as a crude preparation, and the specific bioactive compounds responsible for the observed protective effects were not isolated or structurally characterized. Third, molecular pathways underlying antioxidant enzyme regulation and iNOS inhibition were inferred based on biochemical and histological outcomes rather than confirmed through gene or protein-level signaling assays. Fourth, the study utilized a single, well-characterized batch of *T. claveryi* extract collected from a defined source and time point; given the known influence of geographic origin, seasonal variation, and environmental conditions on fungal phytochemical composition, batch-to-batch variability cannot be excluded and may affect reproducibility. Additionally, the experimental design involved a single dose and treatment duration, which does not allow assessment of dose–response relationships or treatment optimization. Therefore, the present findings should be interpreted as evidence of oxidative stress attenuation and inflammatory damage modulation rather than as proof of therapeutic efficacy. Future research incorporating fractionation, mechanistic assays, and different host models would provide a more comprehensive understanding of the therapeutic potential of *T. claveryi*.

## Conclusion

TCE effectively protects against oxidative stress and inflammatory damage caused by *H. thalassini* infection. Its rich phenolic, flavonoid, and tannin content restores antioxidant enzyme activity, reduces lipid peroxidation, and modulates nitric oxide production, while improving splenic histology and downregulating iNOS expression. These findings highlight TCE’s dual role in neutralizing free radicals and regulating immune responses; however, the observed effects are based on a single, chemically characterized extract batch and may be influenced by natural variability in fungal composition. Therefore, TCE is best viewed as a supportive or complementary intervention rather than a definitive antiparasitic therapy. Future studies should focus on isolating and standardizing the active constituents, evaluating batch-to-batch and seasonal variability, and elucidating the precise molecular mechanisms underlying its protective effects.

## Data Availability

The raw data supporting the conclusions of this article will be made available by the authors, without undue reservation.
